# Editorial for the Special Issue: “Epidemiology, Prognosis and Antimicrobial Treatment of Extensively Antibiotic-Resistant Bacterial Infections”

**DOI:** 10.3390/antibiotics11060804

**Published:** 2022-06-15

**Authors:** Stamatis Karakonstantis, Evangelos I. Kritsotakis

**Affiliations:** 1 Internal Medicine Department, Division of Infectious Diseases, University Hospital of Heraklion, 71500 Heraklion, Greece; 2 Laboratory of Biostatistics, Department of Social Medicine, School of Medicine, University of Crete, 70013 Heraklion, Greece; e.kritsotakis@uoc.gr

The increasing consumption of broad-spectrum antimicrobials is fuelling a vicious cycle leading to extensively drug-resistant (XDR) and pandrug-resistant (PDR) bacteria [1]. Indeed, PDR Gram-negative bacteria (GNB) have been increasingly reported worldwide [2], and are associated with limited treatment options [3] and high mortality [2,4]. The aim of this Special Issue was to collect manuscripts on the epidemiology, prognosis and treatment of XDR/PDR bacterial infections.

The first contribution comes from Gibb J and Wong DW who, based on their literature review, propose a treatment approach for infections by *Stenotrophomonas maltophilia*, a pathogen with intrinsic resistance to many antibiotics (notably carbapenems) [5]. Options for the treatment of *S. maltophilia* resistant to first-line treatments (trimethoprim/sulfamethoxazole, fluoroquinolones, minocycline) are discussed, including cefiderocol (a novel siderophore cephalosporin that is stable against most β-lactamases and can overcome resistance mediated by porin loss and efflux pumps [6]) and the combination of ceftazidime/avibactam with aztreonam (a combination that can overcome resistance mediated by metallo-β-lactamases [7]).

Also within this Special Issue, Losito AR et al. discuss treatment options for difficult-to-treat *P. aeruginosa*, namely: novel β-lactam/β-lactamase-inhibitor combinations (ceftolozane/tazobactam, ceftazidime/avibactam and imipenem/relebactam, as well as combinations in the pipeline), cefiderocol, and fosfomycin-based combinations [8]. Of particular note is that ceftolozane/tazobactam activity may be reduced against *P. aeruginosa* isolates co-resistant to all other anti-pseudomonal β-lactams, as shown by Tantisiriwat W et al. [9]. Moreover, none of the currently available β-lactam/β-lactamase combinations are active against metallo-β-lactamase producing *P. aeruginosa* [3].

Remaining on the topic of *P. aeruginosa*, a potentially underappreciated treatment option is proposed by Ulloa ER and Sakoulas G in this issue, who report the successful use of azithromycin in three cases [8]. Azithromycin is an interesting option, providing an alternative oral treatment against *P. aeruginosa* (fluoroquinolones currently being the only oral option), as well as a potential option against XDR GNB. In vitro studies have demonstrated in media that better reflect in vivo conditions that azithromycin is active against *P. aeruginosa* (as well as other GNB, including *A. baumannii*, Enterobacterales, and *S. maltophilia*) [8,10,11,12]. Animal models further support these in vitro findings [10,12]. Also notable is the potential for synergy with polymyxins, as well as natural cationic antimicrobial peptides [10,12]. However, available clinical data are limited to the case series of this issue [8]. Nevertheless, macrolides have also been used successfully as prophylactic therapy in patients with cystic fibrosis, bronchiectasis, and chronic obstructive pulmonary disease, showing particular benefit in *P. aeruginosa*-colonized patients [13,14,15]. Notably, acquired resistance to macrolides in *P. aeruginosa* has been reported [16], further supporting the notion that macrolides can indeed have direct antibacterial activity against *P. aeruginosa*.

Finally, mechanisms of resistance, heteroresistance, and the in vivo emergence of resistance to cefiderocol have been systematically reviewed [17]. Multiple mechanisms, typically acting in concert, have been identified, including β-lactamases (especially NDM, selected KPC and AmpC variants, OXA-427, and PER- and SHV-type ESBLs), permeability defects (mutations affecting siderophore receptors, porin loss, and the overexpression of efflux pumps) and target modification (PBP-3 mutations). Especially worrisome is the emergence of various β-lactamases, able to cause multi-fold increases in cefiderocol minimum inhibitory concentration, and the high prevalence of heteroresistance. These findings, in combination with reports of in vivo emergence of resistance during treatment, underscore the need for continued surveillance of cefiderocol’s activity, as this agent is being introduced in clinical practice.

The latter publication also highlights that resistance can rapidly emerge against novel antimicrobials as they are being introduced in clinical practice, leaving synergistic combinations as a last-resort treatment option [3]. However, despite numerous in vitro publications and some data from animal models, clinical data on antimicrobial combinations are limited [3,18] and the methodology for identifying clinically relevant antimicrobial combinations needs updating and consensus [19]. Furthermore, the focus on infection prevention and control and antimicrobial stewardship has become even more important in preventing the spread of XDR/PDR pathogens and preserving the activity of new antimicrobials.
